# An Efficient Teager Energy Operator-Based Automated QRS Complex Detection

**DOI:** 10.1155/2018/8360475

**Published:** 2018-09-18

**Authors:** Hamed Beyramienanlou, Nasser Lotfivand

**Affiliations:** Department of Electronic Engineering, Tabriz Branch, Islamic Azad University, Tabriz, Iran

## Abstract

**Database:**

The efficiency and robustness of the proposed method has been tested on Fantasia Database (FTD), MIT-BIH Arrhythmia Database (MIT-AD), and MIT-BIH Normal Sinus Rhythm Database (MIT-NSD).

**Aim:**

Because of the importance of QRS complex in the diagnosis of cardiovascular diseases, improvement in accuracy of its measurement has been set as a target. The present study provides an algorithm for automatic detection of QRS complex on the ECG signal, with the benefit of energy and reduced impact of noise on the ECG signal.

**Method:**

The method is basically based on the Teager energy operator (TEO), which facilitates the detection of the baseline threshold and extracts QRS complex from the ECG signal.

**Results:**

The testing of the undertaken method on the Fanatasia Database showed the following results: sensitivity (Se) = 99.971%, positive prediction (P+) = 99.973%, detection error rate (DER) = 0.056%, and accuracy (Acc) = 99.944%. On MIT-AD involvement, Se = 99.74%, P+ = 99.97%, DER = 0.291%, and Acc = 99.71%. On MIT-NSD involvement, Se = 99.878%, P+ = 99.989%, DER = 0.134%, and Acc = 99.867%.

**Conclusion:**

Despite the closeness of the recorded peaks which inflicts a constraint in detection of the two consecutive QRS complexes, the proposed method, by applying 4 simple and quick steps, has effectively and reliably detected the QRS complexes which make it suitable for practical purposes and applications.

## 1. Introduction

Cardiovascular disease is the primary global cause of death. According to the World Health Organization, about 17.3 million people died of cardiovascular disease in 2008, which represented 30 percent of all global deaths. This number is predicted to grow to more than 23.6 million by 2030 [1, 2]. Heart diseases like cardiovascular disease, sudden death, ischemic heart disease, and cardiac arrhythmias are all diagnosed by analyzing the heart's signal [3–5]. The electrocardiogram is a noninvasive method of recording signals of heart muscle contractions over a period of time. Therefore, the accurate analysis of these signals will result in a more accurate diagnosis of cardiovascular diseases [6, 7]. An ECG signal is a combination of QRS complex, P and T peaks, and sometimes includes U peak. The detection of the QRS complex also helps to detect and determine P and T peaks, QT interval, ST interval, and the respiratory rate, which are considered as the human's vital signs. Therefore, the accurate recognition of QRS complex has a significant role in the accurate diagnosis of heart disease [8].

During the past decades, a variety of QRS complex detection methods have been developed [9] such as Pan–Tompkins method of R-wave detection [10], support vector machine [11], and the wavelet method, which is an analytical technique based on time-frequency chromatography. The wavelet transform is widely used in medical signal analysis such as EEG or ECG. However, this has a drawback because by applying a fixed scale, the signal characteristics are ignored [3, 12, 13]. Kalman filters use a dynamic model derived from a dynamic system to predict the hidden state in a nonlinear approach [14]. Artificial neural networks are an ideal self-correcting nonlinear process used in a wide range of tasks [15]. Shannon energy computes the average signal energy in a signal spectrum. In other words, it reduces the high intensity to balance out with the low intensity [16–18]. The Adaptive Double Threshold Filter (ADTF) and Discrete Wavelet Transform (DWT) are used to reduce the noise in the ECG signal to improve the ECG signal filtering [19]. Hermit transformation, which is used as an alternative to the Fourier transformation, may by optimization, shows an improved performance [20]. Teager Energy Operator (TEO) mainly shows the frequency and instantaneous changes of the signal amplitude that is very sensitive to subtle changes. Although TEO was first proposed for modeling nonlinear speech signals, it was later widely applied in the audio signal processing. Using TEO can minimize the effects of P and T waves on QRS complex detection [21]. Remarkable research efforts have been developed to analyze the sensitive point of the ECG signal based on TEO [22, 23].

The aim of this study is to propose a new approach based on an innovative viewpoint using TEO to detect QRS complex in the ECG signal. The recorded ECG signal may be affected by noise interference, such as power line interference, which must be eradicated for more accuracy. The Section 2 of the study consists of a series of preprocessing measures to minimize the noises before QRS complex detection on the ECG signal. This includes low-pass filter which removes noises such as power line interference. Since P or T peaks may interfere with the TEO computation, the moving average technique is used to smoothen and envelope the spikes in the signal. Sensitivity, positive prediction, and accuracy of the proposed algorithm from Fantasia Database, MIT-AD, and MIT-NSD are evaluated in the Section 3 of this article. Finally, in Sections 4 and 5 of the study, a discussion and conclusions are presented.

## 2. Methodology

**Figure 1:**
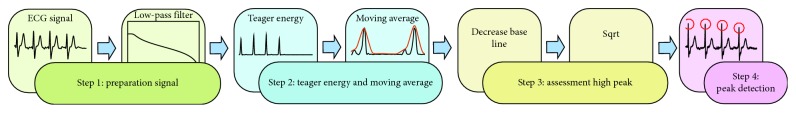
The diagram block shows 4 steps of QRS complex detection.

The details of the proposed method are illustrated in Figure 1. The QRS complex detection procedure involves four steps.

(i) Most of the time, the recorded ECG signal is afflicted by noises [24]. The noise frequencies generated by the power lines' interferences are in the range of 50 to 60 Hz. The noises generated by the muscle contractions and the electrodes placed on the body skin are in the range of 38 to 48 Hz. These greatly impede on the ECG signals. However, a notch filter is very effective in removing these noises. The maximum density of the QRS complex is between 5 to 20 Hz [17, 25]. Therefore, the IIR Butterworth digital filter is the best compromise for phase response and signal attenuation. It has no ripple in the band-pass and is more efficient than the FIR filter [26, 27]. To reduce the noises from the electrical (device) components in order to make a peak clearer for detection, a Butterworth low-pass digital filter, with order 4 and cut-off frequency of 15 Hz, was used.
(ii) TEO has various applications, especially in AM and FM signal processing such as speech signals. TEO can be driven from a second-order differential equation [28]. The total energy of oscillation (i.e., the sum of kinetic and potential energies) can be obtained from the following equation:
  (1) $$\begin{array}{c} E=\frac{1}{2}kx^{2}+\frac{1}{2}mx^{2}, \end{array}$$
where *m* is the mass of the oscillating body and *k* is the spring constant.
Using the formula in (1), a periodic harmonic formula can be obtained:
  (2) $$\begin{array}{c} x\left(t\right)=A\cos\left(\Omega t+\phi \right), \end{array}$$
where *ϕ* is the phase shift, *Ω* is the oscillation frequency, *A* is the oscillation amplitude, and *x*(*t*) denotes the position of the oscillating body with respect to time. Using (1) and (2), the essential harmonic energy to generate signals can be calculated:
  (3) $$\begin{array}{c} E=\frac{1}{2}mA^{2}\alpha A^{2}\Omega^{2}. \end{array}$$
The following is a simplified form of TEO:
  (4) $$\begin{array}{c} \Psi_{\text{c}}\left[x\left(t\right)\right]={\left[\frac{d}{dt}x\left(t\right)\right]}^{2}-x\left(t\right)\frac{d^{2}}{dt^{2}}x\left(t\right),\\ \Psi_{\text{c}}\left[x\left(t\right)\right]={\left[x^{\prime }\left(t\right)\right]}^{2}-x\left(t\right)x^{\prime \prime }\left(t\right). \end{array}$$
Substituting *nT* for *t* we will get the following equation:
  (5) $$\begin{array}{c} \Psi_{\text{d}}\left(x\left[n\right]\right)=x^{2}\left[n\right]-x\left[n-1\right]x\left[n+1\right], \end{array}$$
where Ψ_c_[*x*(*t*)] is the energy operator for continuous time *t*, *x*(*t*) is the *t* ^th^ signal component, [*x*′(*t*)] and [*x* ^″^(*t*)] are the first and second derivatives of *x*(*t*), respectively, *T* is the sample period, and *n* is the sample size [28, 29].
The dynamicity of the heart beats creates an intermittent and nonlinear pattern for TEO. Since TEO itself is a nonlinear operator, it nonlinearly captivates the intermittent characteristics.
(iii) After computing TEO, in some signals, spikes of energies are observable and are attributed to P and T peaks in QRS complex. Although not wide in range, they hamper the accurate detection of the QRS complex. To resolve the situation, these spikes should be converted into energy envelopes. There are several methods for this such as Hilbert transform [6] or averaging method [30]. In the present study, moving average the following equation is used:
  (6) $$\begin{array}{c} \text{MA}=\text{filter}\left(h,j,\text{TEO}\right). \end{array}$$
Here *h* defines a rectangle with *L* length, *j* is a constant and is equal to 1, and TEO defines Teager energy from previous steps. To increase the small amplitude, square root is used:
  (7) $$\begin{array}{c} S=\text{sqrt}\left(\text{MA}\right). \end{array}$$
Here MA is the moving average obtained from the previous step.
To decrease the baseline signal's value below zero, the following formula is used [16]:
  (8) $$\begin{array}{c} \text{BD}=\frac{x\left[n\right]-\mu }{\sigma }, \end{array}$$
  where *σ* is the standard deviation and *μ* is defined as signal average.
(iv) The process of peak detection includes the following step:
  (9) $$\begin{array}{c} \left\{\begin{array}{cc} \text{signal}\left[n\right], & \text{baseline}<\text{signal}\left[n\right],\\ \text{not}\;\;\mathrm{select}, & \text{signal}\left[n\right]<\text{baseline,} \end{array}\;\right.n=2,3,4,\ldots , \end{array}$$
  where baseline (0) is the threshold level for peak detection and R peaks are found in an ECG signal by searching the maximum peak within ±50 samples (length of window = min (RR interval)) of the recognized location of the candidate R peak in the previous step (Equation (9)).

## 3. Results

Under the supervision of the National Research Center, the PhysioBank database was developed by the National Institute of Health in order to do a clinical diagnosis and conduct research on complex cardiovascular physiologic signals [31]. The proposed method was tested on three different ECG databases [32] including Fantasia Database (FTD), MIT-BIH Arrhythmia Database (MIT-AD), and MIT-BIH Normal Sinus Rhythm Database (MIT-NSD).

The suggested peak detection method based on TEO has been implemented with MATLAB R2016a on a minimum laptop with a 4 GB of memory and Intel core i3-4000M 2.4 GHz CPU on Windows 10. This algorithm takes less than 0.026 second.

The following formulae were used to determine the performance, sensitivity, error rate detection, positive prediction, and the accuracy of the proposed method:

(10) $$\begin{array}{c} \text{Sensitivity }\left(\text{Se}\right)=\frac{\text{TP}}{\text{TP}+\text{FN}}\times 100\%,\\ \text{Detection}\;\;\mathrm{error}\;\;\mathrm{rate}\;\left(\text{DER}\right)=\frac{\text{FP}+\text{FN}}{\text{TP}}\times 100\%,\\ \text{Positive}\;\;\mathrm{predictivity}\;\;\mathrm{rate}\;\left(+\text{P}\right)=\frac{\text{TP}}{\text{TP}+\text{FP}}\times 100\%,\\ \text{Accuracy }\left(\text{Acc}\right)=\frac{\text{TP}}{\text{TP}+\text{FP}+\text{FN}}\times 100\%. \end{array}$$

TP is the number of R peaks, FN is the number of missed R peaks, and FP is the false positive prediction of R peak due to the existing noise with dispositioned true R peak.

### 3.1. MIT-BIH Arrhythmia Database

**Figure 2:**
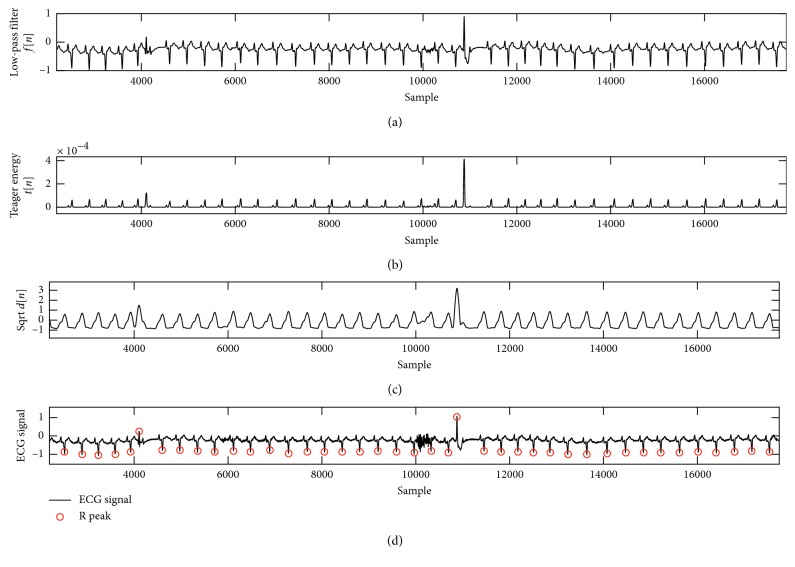
Recognition of R peaks in 108 records. The *Y*-axis represents amplitude, and *X*-axis represents the samples.

**Figure 3 fig3:**
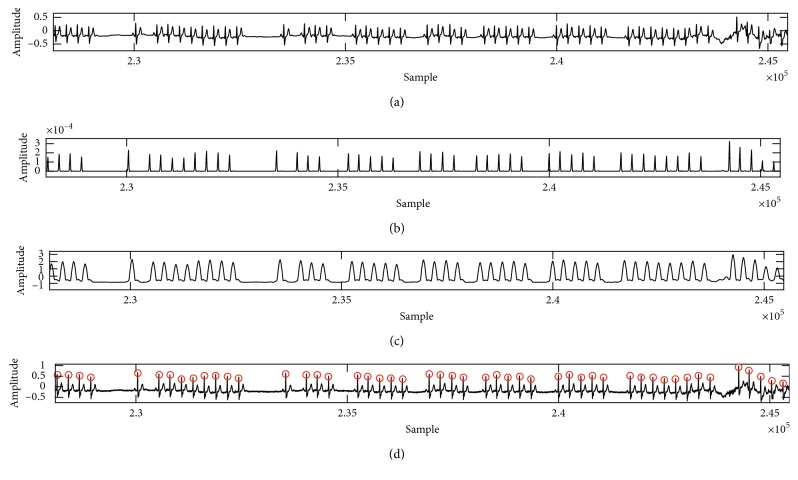
(a) Low-pass filter, (b) Teager energy operator, (c) moving average, and (d) recognition of R peaks in 232 record. The *Y*-axis represents amplitude, and *X*-axis represents the samples.

**Figure 4:**
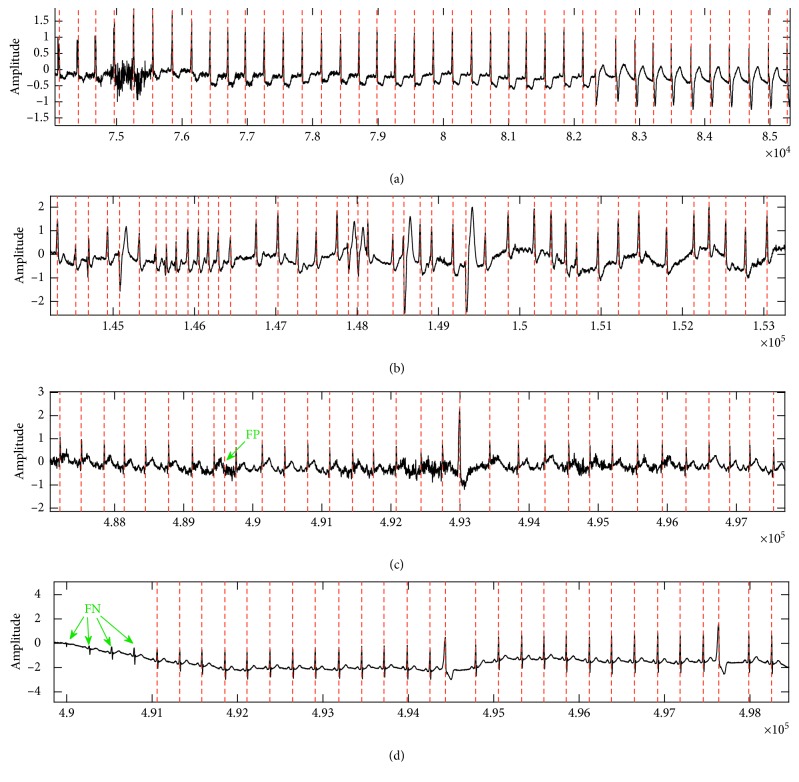
Examples of the detected QRS Complex from various cases. (a) 104, (b) 203, (c) 228, and (d) 116. The *Y*-axis represents amplitude, and *X*-axis represents the samples.

MIT-AD contains slightly over 30 minutes of recordings in 48 records. The sampling frequency was set to 360 Hz with 11-bit ADC resolution. The subjects who were chosen for this study were 22 women aged 23 to 89 years and 25 men aged 32 to 89 years [31, 33].  Table 1 depicts the details of detected QRS complex in channel 1. The results showed that sensitivity was at 99.74% with a 0.391% (detection) error and 99.97% positive prediction with an accuracy of 99.71%.  Table 2 compares the proposed algorithm in this study with those of other studies. All the stages and the process of QRS detections are illustrated in Figures 2 [Fig fig3]–4. The MIT-AD is available on [36].

In Figures 2 and 3, (a) reveals wandering signals. (b) shows that after calculating Teager energy, the amplitudes of the signals are very low and close to zero. Therefore, small values with low energy are reduced to zero, and the wandering signals (drift) are canceled.

### 3.2. Fantasia Database

Fantasia Database (FTD) contains 40 cases in both groups: the young group aged 21 to 34 years (f1y01 … f2y10 and f2y01 … f2y10) and the elderly group aged 68 to 85 years (f2o01 … f2o10 and f2y01 … f2y10), with an average of 5 men and 5 women in each group. The members of each group underwent 120 minutes of continuous supine resting with complete care. The sampling frequency was set at 250 Hz, with a 16- and 12-bit resolutions for ADC. The records included 2 or 3 channels, such as respiration, ECG signal, and blood pressure [31, 37]. Fantasia Database is available on [38].

**Figure 5:**
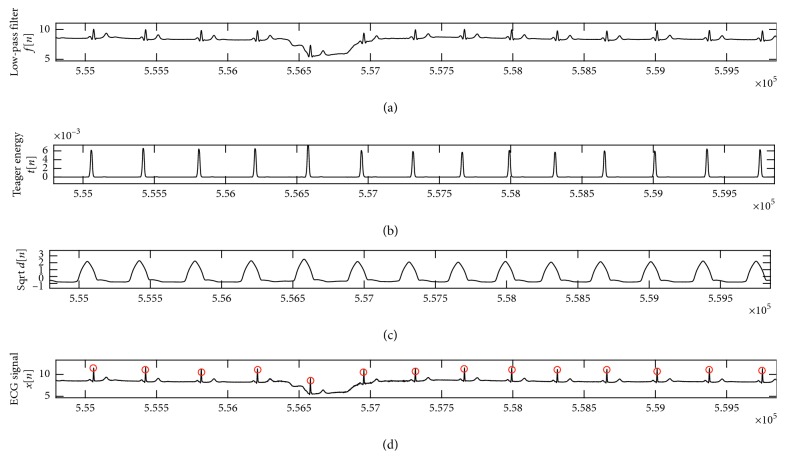
The phases of QRS complex detection in f1o09 case. (a) Low-pass filter phase; (b) the Teager energy from the Equation (5); (c) the moving average; (d) the final detection of QRS complex on ECG signal. The *Y*-axis represents amplitude, and *X*-axis represents the samples.

**Figure 6:**
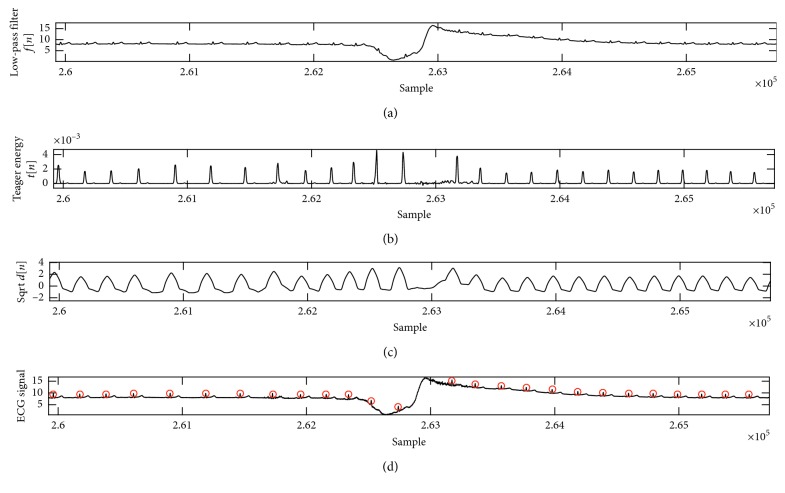
Signal processing steps of the proposed R-peak detector using the case f1y06m. (a) The recognition phases by applying a low-pass filtering, (b) the Teager energy, (c) decreased baseline after moving average and sqrt, and (d) the detection phases. The *Y*-axis represents amplitude, and *X*-axis represents the samples.

The QRS complex detection details for the channel 2 in Fantasia Database are presented in Table 3. Here too, the results showed 99.971% sensitivity with 0.056% detection error, and 99.973% positive prediction with an accuracy of 99.944%.  Table 4 shows the comparison of the proposed method with the other studies. Figures 5 and 6 illustrate another example of detection: QRS Complex in the Fantasia Database with both elderly and young subjects. As shown in this figure, the proposed method can remove drift noise and detect correct location beat.

### 3.3. MIT-BIH Normal Sinus Rhythm Database

**Figure 7:**
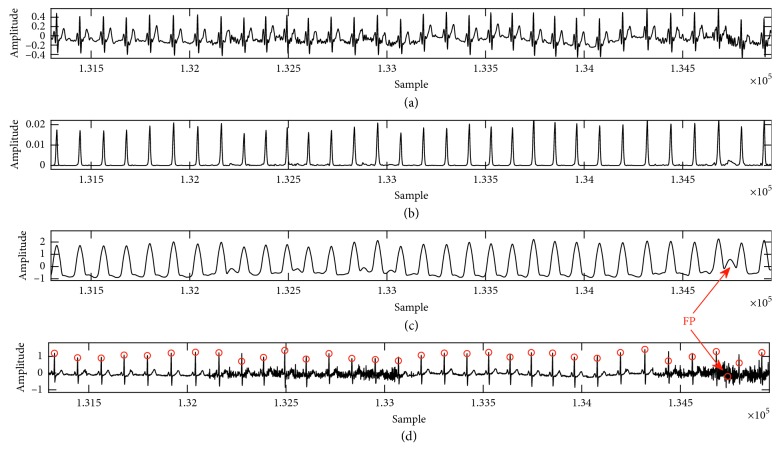
Signal processing steps of the proposed R-peak detector using case 16272 with Gaussian white noise. (a) Low-pass filter; (b) the Teager energy; (c) moving average for eliminated spikes and BD stage; (d) detection of R peak. FP is false-positive prediction when the noise is detected. The *Y*-axis represents amplitude, and *X*-axis represents the samples.

MIT-NSD contains 18 long-term two-channel ECG recordings. This database includes 5 men, aged 26 to 45 and 13 women, aged 20 to 50. Frequency sampling equals to 128 Hz with 12-bit ADC resolution [31]. The details of QRS complex detection of channel 1 of MIT-NSD is presented in Table 5. The obtained values showed that sensitivity was equal to 99.878%, with an error equal to 0.134, positive prediction was equal to 99.989%, and accuracy was equal to 99.867%.  Table 6 includes a comparison of the proposed algorithm with the other studies.  Figure 7 illustrates the QRS detection in record with Gaussian white noise. As shown in the figure, the proposed method removed Gaussian white noise, but T peak was detected as a beat. MIT-NSD Database is available on [39].

## 4. Discussion

The aim of the present research is to use a novel algorithm based on the Teager energy operator in ECG signal to detect QRS complex. The main findings of the study indicated the high reliability and accuracy of this method in QRS complex detection. In spite of applying zero-phase digital filter to maintain QRS complex location, the zero-phase filter is anticausal, and the results showed that the present method had faced a little lag which was less than 0.026 second. Only a detection shift of less than 0.05 second is acceptable [40].

In testing the present method on MIT-AD, some records such as 203 and 210 are main sources of error. The error rate is higher than 1%, which is equal to 0.291. Record 203 has a great number of QRS complexes with multiform ventricular arrhythmia. The TEO phase revealed that the amplitudes are very low and close to zero. Due to this fact, the present method indicated quite a weak performance about records: 203, 19090, and 19830. Records 230, 114, 113, 107, and 106 contain high and sharp T peaks. Record number 207 includes some ventricular flutter (VF) intervals. Those intervals are not interpreted and they go out of studies. One of the constraints of the proposed method is when the QRS complex locations are very close to each other. The length (*L*) of moving average of phase 2 is assumed to be about 0.17  ×  *fs*. The recorded signals of Fantasia Database included a variety of cardiac morphology, heart failures, and noises from sources like power lines, white Gaussian noise, and flicker noise(1/*f*). Lowering the baseline is the main factor contributing to R-peak losses in the MIT-BIH Normal Sinus Rhythm Database.

The advantages of the proposed method are a reduced number of steps to implement, no need for an excessive memory capacity or learning stage, a fast method of detection, a set baseline threshold, and no complex mathematical relationships.

## 5. Conclusion

The present study detects QRS complex based on Teager energy, which was tested on four databases. It is a novel algorithm with an acceptable accuracy for ECG baseline prediction. The obtained results from testing the presented method on the Fantasia Database involved: sensitivity (Se) = 99.971%, positive prediction (+P) = 99.973%, detection error rate (DER) = 0.056%, and accuracy (Acc) = 99.944%. On MIT-AD involvement, Se = 99.74%, +P = 99.97%, DER = 0.291%, and Acc = 99.71%. On MIT-NSD involvement, Se = 99.878%, +P = 99.989%, DER = 0.134%, and Acc = 99.867%. The provided results indicate that the presented method is reliable to detect QRS complex, and because the relationships are simple, the proposed method has a better performance than other sophisticated techniques such as neural networks. The results show that the proposed method is simple, effective, accurate, and suitable for practical application. To avoid the lag from zero-phase filter, a low-pass filter and a moving average were used, but still, the signal faced a shift that was about 0.026 s.

## Figures and Tables

**Table 1:** Results of QRS detection in MIT-BIH Arrhythmia Database (MIT-AD).

Case	TP + FN	TP	FN	FP	DER%	Se%	P+	Acc%	Time (s)
100	2273	2272	1	0	0.044	99.956	100.000	99.956	0.842011
101	1865	1866	0	1	0.054	100.000	99.946	99.946	0.831718
102	2187	2187	0	0	—	100.000	100.000	100.000	0.800651
103	2084	2083	1	0	0.048	99.952	100.000	99.952	0.832546
104	2229	2232	0	3	0.134	100.000	99.866	99.866	0.819847
105	2572	2584	12	4	0.619	99.538	99.845	99.385	0.821659
106	2027	2023	4	0	0.198	99.803	100.000	99.803	0.826349
107	2137	2134	3	0	0.141	99.860	100.000	99.860	0.831685
108	1763	1758	5	0	0.284	99.716	100.000	99.716	0.828862
109	2532	2527	5	0	0.198	99.803	100.000	99.803	0.839979
111	2124	2123	0	0	—	100.000	100.000	100.000	0.808473
112	2539	2539	0	0	—	100.000	100.000	100.000	0.822048
113	1795	1794	1	0	0.056	99.944	100.000	99.944	0.818563
114	1879	1856	23	0	1.239	98.776	100.000	98.776	0.80345
115	1953	1953	0	0	—	100.000	100.000	100.000	0.818539
116	2412	2389	23	0	0.963	99.046	100.000	99.046	1.321399
117	1535	1535	0	0	—	100.000	100.000	100.000	0.795675
118	2278	2279	0	1	0.044	100.000	99.956	99.956	0.082806
119	1987	1988	0	0	—	100.000	100.000	100.000	0.827755
121	1863	1862	1	0	0.054	99.946	100.000	99.946	0.804042
122	2476	2476	0	0	—	100.000	100.000	100.000	0.833951
123	1518	1517	0	0	—	100.000	100.000	100.000	0.816707
124	1619	1617	2	0	0.124	99.876	100.000	99.876	0.832643
200	2601	2601	0	0	—	100.000	100.000	100.000	0.79576
201	1963	1952	11	0	0.564	99.440	100.000	99.440	0.805514
202	2136	2116	19	0	0.898	99.110	100.000	99.110	0.810542
203	2980	2911	69	0	2.370	97.685	100.000	97.685	0.805071
205	2656	2653	3	0	0.113	99.887	100.000	99.887	0.818077
207	1860	1863	1	3	0.215	99.946	99.839	99.786	0.809665
208	2955	2935	20	0	0.681	99.323	100.000	99.323	0.814803
209	3005	3008	0	3	0.100	100.000	99.900	99.900	0.789794
210	2650	2628	22	2	0.913	99.170	99.924	99.095	0.813432
212	2748	2748	0	0	—	100.000	100.000	100.000	0.797922
213	3251	3243	8	0	0.247	99.754	100.000	99.754	0.826331
214	2262	2256	6	0	0.266	99.735	100.000	99.735	1.04979
215	3363	3358	5	0	0.149	99.851	100.000	99.851	0.796735
217	2208	2205	3	0	0.136	99.864	100.000	99.864	0.816972
219	2154	2154	0	0	—	100.000	100.000	100.000	1.032321
220	2048	2047	1	0	0.049	99.951	100.000	99.951	0.806677
221	2427	2423	4	0	0.165	99.835	100.000	99.835	0.817536
222	2483	2475	8	0	0.323	99.678	100.000	99.678	0.837608
223	2605	2594	11	0	0.424	99.578	100.000	99.578	0.79572
228	2053	2065	1	12	0.630	99.952	99.422	99.374	0.808852
230	2256	2256	0	0	—	100.000	100.000	100.000	0.806291
231	1571	1571	0	0	—	100.000	100.000	100.000	0.815568
232	1780	1786	0	4	0.224	100.000	99.777	99.777	0.802255
233	3079	3070	9	0	0.293	99.708	100.000	99.708	0.795327
234	2753	2750	3	0	0.109	99.891	100.000	99.891	0.80702
Total	109494	109262	285	33	0.291	99.740	99.970	99.710	0.81952

**Table 2:** Comparison of performance of our proposed method with other methods using MIT-BIH Arrhythmia Database (MIT-AD).

Ref.	Method	DER	Se	P+	Acc%
[10]	Low-pass filtering, high-pass filtering derivative filtering	0.675	99.762	99.565	99.329
[34]	Multiscale mathematical morphology	0.0039	99.81	99.80	99.621
[35]	Shannon energy envelope, Hilbert transform	0.205	99.93	99.86	99.79
[8]	Median filter, Savitzky–Golay, Kurtosis	0.93	99.50	99.56	99.08
[17]	Shannon energy	0.164	99.95	99.88	99.84
[18]	Wavelet transform, Shannon energy envelope	0.163	99.93	99.91	99.838
Proposed method	Teager energy operator	0.291	99.74	99.97	99.71

**Table 3:** Results of QRS detection in Fantasia Database (FTD).

Case	TP	FN	FP	DER%	Se%	P+	Acc%	Time (s)
f1o01m	3988	0	0	—	100.000	100.000	100.000	1.051548
f1o02m	3813	0	0	—	100.000	100.000	100.000	1.040927
f1o03m	4046	0	0	—	100.000	100.000	100.000	1.055944
f1o04m	3433	0	3	0.087	100.000	99.913	99.913	1.02246
f1o05m	3720	2	4	0.161	99.946	99.893	99.839	0.997257
f1o06m	3408	0	0	—	100.000	100.000	100.000	1.020774
f1o07m	4025	0	0	—	100.000	100.000	100.000	1.031825
f1o08m	4739	0	3	0.063	100.000	99.937	99.937	1.012155
f1o09m	2796	0	2	0.072	100.000	99.929	99.929	1.016363
f1o10m	4602	0	0	—	100.000	100.000	100.000	1.030248
f1y01m	4917	0	0	—	100.000	100.000	100.000	1.026809
f1y02m	3967	0	0	—	100.000	100.000	100.000	1.029854
f1y03m	4289	0	0	—	100.000	100.000	100.000	1.006034
f1y04m	2998	0	0	—	100.000	100.000	100.000	1.005517
f1y05m	3942	0	4	0.101	100.000	99.899	99.899	1.015047
f1y06m	3906	1	5	0.154	99.974	99.872	99.847	1.004932
f1y07m	3381	0	1	0.030	100.000	99.970	99.970	1.009606
f1y08m	4098	0	0	—	100.000	100.000	100.000	1.043768
f1y09m	4509	0	2	0.044	100.000	99.956	99.956	1.029059
f1y10m	4912	1	0	0.020	99.980	100.000	99.980	1.046556
f2o01m	4216	0	0	—	100.000	100.000	100.000	1.026018
f2o02m	3594	5	0	0.139	99.861	100.000	99.861	1
f2o03m	3765	1	0	0.027	99.973	100.000	99.973	1.063687
f2o04m	3857	0	0	—	100.000	100.000	100.000	1.006415
f2o05m	4926	7	4	0.223	99.858	99.919	99.777	1.014471
f2o06m	2987	0	1	0.033	100.000	99.967	99.967	1.024759
f2o07m	3373	0	0	—	100.000	100.000	100.000	1.066044
f2o08m	4151	0	0	—	100.000	100.000	100.000	1.520268
f2o09m	3335	1	1	0.060	99.970	99.970	99.940	1.037157
f2o10m	4996	2	1	0.060	99.960	99.980	99.940	1
f2y01m	4586	0	0	—	100.000	100.000	100.000	1.016468
f2y02m	2807	0	0	—	100.000	100.000	100.000	1.018488
f2y03m	3882	1	0	0.026	99.974	100.000	99.974	1.023054
f2y04m	4943	0	7	0.142	100.000	99.859	99.859	1.038463
f2y05m	5169	2	0	0.039	99.961	100.000	99.961	1
f2y06m	4017	0	0	—	100.000	100.000	100.000	1.017889
f2y07m	3717	0	0	—	100.000	100.000	100.000	1.031076
f2y08m	4014	4	3	0.174	99.900	99.925	99.826	1
f2y09m	4870	11	2	0.267	99.775	99.959	99.734	1
f2y010m	4032	8	1	0.223	99.802	99.975	99.777	1
Total	160726	46	44	0.056	99.971	99.973	99.944	0.86252

**Table 4:** Comparison of the proposed method with other methods using Fantasia Database (FTD).

	DER%	SE%	+P%	Acc%
Sharma and Sunkaria [8]	0.19	99.90	99.91	99.81
Proposed method	0.056	99.971	99.973	99.944

**Table 5:** Results of QRS detection in MIT-BIH Normal Sinus Rhythm Database (MIT-NSD).

Case	TP	FN	FP	DER%	Se%	+P%	Acc%	Time (s)
16265	11497	1	0	0.009	99.991	100.000	99.991	1.035
16272	7992	2	7	0.113	99.975	99.912	99.888	1.3
16273	10431	2	0	0.019	99.981	100.000	99.981	1.01
16420	10687	20	2	0.206	99.813	99.981	99.795	1.3
16483	12157	5	0	0.041	99.959	100.000	99.959	1.02
16539	9130	7	8	0.164	99.923	99.912	99.836	1.04
16773	9679	1	0	0.010	99.990	100.000	99.990	1.04
16786	9510	2	0	0.021	99.979	100.000	99.979	1.029
16795	10386	0	0	—	100.000	100.000	100.000	1.31
17052	8851	4	0	0.045	99.955	100.000	99.955	1.049
17453	11258	0	1	0.009	100.000	99.991	99.991	1
18177	11907	5	0	0.042	99.958	100.000	99.958	1.32
18184	10888	13	1	0.129	99.881	99.991	99.872	1.023
19088	12360	3	0	0.024	99.976	100.000	99.976	1.04
19090	10481	65	1	0.630	99.384	99.990	99.374	1.33
19093	9111	0	0	—	100.000	100.000	100.000	1.028
19140	11316	39	0	0.345	99.657	100.000	99.657	1.03
19830	14811	66	2	0.459	99.556	99.986	99.543	1.047
Total	192452	235	22	0.134	99.878	99.989	99.867	1.108389

**Table 6:** Comparison of the proposed method with other methods using MIT-BIH Normal Sinus Rhythm Database (MIT-NSD).

Method	DER%	SE%	+P%	Acc%
Sharma and Sunkaria [8]	1.21	99.36	99.43	98.81
Proposed method	0.134	99.878	99.989	99.867

## Data Availability

The data used to support the findings of this study are available in [36, 38, 39].
